# A Treatise on General Pathology

**Published:** 1853-07

**Authors:** 


ART. V.—*A Treatise on General Pathology.* By Dr. J. HENLE, Professor of Anatomy and Physiology, in Heidelberg. Translated from the German, by HENRY C. PRESTON, A. M., M. D. Philadelphia: Lindsay & Blackiston. 1853.

The science of General Pathology is so vast in its intimate relations with every department of medicine, is so essential to all true inference or reasoning upon diseased conditions of the animal economy, and is so necessary to the thorough equipment of the philosophical practitioner, that we hail with pleasure every new contribution to the stores of the science. The author of the book before us is a master spirit in general pathology, and he will command the respect and admiration of all who place themselves under his instruction. His work is not only instructive, but is entertaining, and cannot fail to interest, at least in its historical features.

Dr. Henlé commences his labors with a review of medical systems, in which he presents the claims of rational pathology in a striking light. His symptommatology and nosology, contained in the divisions of rational pathology cannot fail to command respect.

The history of medical systems is divided by Dr. Henlé into seven divisions, and we strongly urge these historic details upon the attention of the reader. We are restricted, by want of space, to a single quotation, in this department of the work :

"The distingnishing character of Rokitansky's school consists in its scientific perception and application of these relations. Its reputation was still farther increased, when, by the quantitative chemical analyses of the blood, most thoroughly made by Denis and La Canu, it had become possible to ascertain the abnormality of the blood chemically. Rokitansky had, as it were, already prepared the departments under which this accession to the material was to be brought. Andral and Gavarret are indebted to him for the effect which their work upon the pathology of the blood excited in Germany, still more than in France.

But with all these enrichments of the science, the school remained an anatomical one, and did not get rid of those fundamental errors which had misled the solid pathological anatomists upon the barren heath of speculation. Nothing was changed, except that the unity of disease forsook its old quarters in the organs and tissues, and took up new ones in the blood. It was no more inflammation, but excess of fibrin ; no more nervous fever, nor even gastro-enteritis, but albuminous crasis. There were no investigations made concerning the development of symptoms from the dyscrasy, nor of the development of dyscrasia from external causes ; but, worse than this, flimsy dogmatical hypotheses were adduced concerning both. That in Vienna, under such circumstances, limited to the result of post-mortem examinations, the school could have remained at this stage of investigation, is conceivable. But it seems more strange that an Andral should have presumed to explain, with a similar alteration of the blood, so hetrogeneous and complicated diseases as typhus, small-pox, scarlatina, and many others.

Let us turn to England, where, as remarked above, since the time of Harvey all inquiries into organic nature were answered experimentally. John Hunter was the first who transferred this method to the proper department of pathology. His work upon the blood, inflammation, and gun-shot wounds reveals a series of genuine natural scientific investigations concerning one of the most important morbid processes. It includes the microscopic observations of Leeuwenhoek concerning the circulation, and those of Hewson concerning the blood-corpuscles, and adds, moreover, the investigations of Hastings, Thompson, and others. In Germany, the school of Dollinger first investigated this fruitful subject, and notwithstanding it came to conclusions which are now proved for the most part erroneous, still at that time it had established its principles so securely, that we cannot again entirely lose sight of it.

Bell investigated pathology in another way. The physiology of the nerves advanced directly with the pathology of the nerves, after the idea had been conceived of creating artificial morbid condition, which might explain the importance of individual structures. From that time almost every new physiological fact served for the elucidation of pathological phenomena. The investigations of Flourens concerning the function of the individual parts of the brain, and Marshall Hall's theory of reflex action form epochs, the former as the foundation of a future rational pathology of the soul, the latter on account of its influence upon the doctrine of nervous sympathies.

Many similar acquisitions of modern times might be noticed, but we approach the present contents of our science, which it is the intention of this book to demonstrate. Duty will not allow me here to omit the name of one man, the physician who first in Germany, and with German fidelity, applied physiological experiments to the critical examination of general pathological opinions; I mean Stieglitz, whose pathological investigations (published in 1832) shall remain worthy of imitation on account of their method, when their substance shall have long grown obsolete.

One point more, I think, ought to be rendered conspicuous. I have shown how the pathologico-anatomical tendency was completed by means of a chemical element, and came partly in collision with it. Pathological physiology has already proceeded in the same way. If there, as it were a dumb chemical abnormality, was supposed instead of the morbific unity, so here, a fluid, chemical process is recognised as the cause of morbid symptoms. Organic chemistry has become physiological chemistry. The origin of this change lies in the discoveries of Mul der, whereby the identity of the vegetable and animal sperm, and the common basis of all spermatic affinities, was proved. Thereby only was a theory of nutrition possible, which, on the one side, limited legitimately the faculty ascribed to the animal of forming the substances of its body; on the other side, investigated the means whereby metamorphoses of the material substances are accomplished. By the recognition of those most general fundamental principles, the physiology of the reciprocal change of matter has already gained a new and more positive form. Pathology also should promise itself abundant gain from a considerate application of those principles to the explanation of morbid processes.—Would that the fruits brought to maturity in sudden heat might not hasten to a speedy decay; and, above all, would that they might not be abused to decoy us back aga n into the old fetters. Already in the school of Liebig, which is as ignorant of modern improvements as of the earlier errors of our science, medicine is again metamorphosed into one of those crude humoral theories which, spreading like contagion, have hitherto followed after each chemical discovery. Not even the teleological vital force would be left to us; because Liebig understands, as well as his predecessors, how to draw out from the fine tissue of organic life a few threads, in the shape of chemical processes, and then to offer us the tangled skein as the share of vitalism. A host of weak eclectics praise him, that they are once more permitted to establish themselves in their inherited ideas, which they had feared they must abandon. In opposition to this reaction, which, like every reaction, obtrudes itself violently and vehemently, and in case of necessity renders contradicting facts suspicious, or silently suppresses them, physiology is obliged to take our threatened improvements under its protection. With the inborn legitimacy of facts, physiology shall encounter the couservatism which usurps opinion; but history shall teach us to mistrust every theory, which, without paying attention to the symptoms of diseases, has the impudent boldness to express their nature with one comprehensive word."

The department of General Pathology is divided into the following subjects: *Definition and nature of disease*

—*general etiology—disease in its relations of extent—the relations of disease with regard to time.* These various themes are handled in the very spirit of a master of the whole subject, and we commend Dr. Henlé to our readers as one eminently entitled to their respect, and most careful study.

